# MmoSTI restriction endonuclease, isolated from *Morganella morganii* infecting a tropical moth, *Actias selene*, cleaving 5′-|CCNGG-3′ sequences

**DOI:** 10.1007/s13353-015-0308-3

**Published:** 2015-08-18

**Authors:** Marta A. Skowron, Joanna Zebrowska, Grzegorz Wegrzyn, Piotr M. Skowron

**Affiliations:** Department of Molecular Biology, Division of Biology, University of Gdansk, Wita Stwosza 59, 80-308 Gdansk, Poland; Department of Molecular Biotechnology, Institute for Environmental and Human Health Protection, Division of Chemistry, University of Gdansk, Wita Stwosza 63, 80-308 Gdansk, Poland

**Keywords:** *Actias selene*, Interrupted palindrome, *Morganella morganii*, Restriction endonuclease

## Abstract

A type II restriction endonuclease, MmoSTI, from the pathogenic bacterium *Morganella morganii* infecting a tropical moth, *Actias selene*, has been detected and biochemically characterized, as a potential etiological differentiation factor. The described REase recognizes interrupted palindromes, i.e., 5′-CCNGG-3′ sequences and cleaves DNA leaving 5-nucleotide (nt) long, single-stranded (ss), 5′-cohesive ends, which was determined by three complementary methods: (*i*) cleavage of custom and standard DNA substrates, (*ii*) run-off sequencing of cleavage products, and (*iii*) shotgun cloning and sequencing of bacteriophage lambda (λ) DNA digested with MmoSTI. MmoSTI, the first 5′-CCNGG-3′ REase characterized from *M. morganii,* is a neoschizomer of ScrFI, which cleaves DNA leaving 1-nt long, ss, 5′-cohesive ends. It is a high-frequency cutter and can be isolated from easily cultured bacteria, thus it can potentially serve as a tool for DNA manipulations.

## Introduction

Type II REases recognize defined DNA sequences and cleave within or in close, defined vicinity of this particular sequence. These enzymes, in the form of monomers, dimers or tetramers, typically act independently of the associated methyltransferase. They cut DNA generating either ‘blunt’ double-stranded DNA ends or cohesive ss and almost all of them require Mg^2+^ ions as cofactors (Loenen et al. 2014). There are both enzymes which recognize symmetric and asymmetric sequences, whereas interrupted palindromes such as GC|TNAGC recognized by EspI (Calléja et al. 1984) are also considered symmetric.

Type II REases are ubiquitous tools in recombinant DNA technology; numerous applications of these enzymes are exemplified by: restriction analysis of DNA fragments and entire genomes, molecular cloning, ‘Southern blot’, constructing protein production systems, DNA amplification methods, studies on DNA-protein interactions, genetic disease analysis (RFLP and other methods), taxonomy, crime detection, molecular archaeology, creating transgenic plants and animals (Roberts 2005). To meet the demands of biotechnology and expanding research techniques, new specificities are needed for scientific and commercial usage. As of 06/05/2015, only 363 different prototype specificities have been described biochemically or genetically (REBASE; Roberts 2014), out of tens of thousands statistically possible combinations of 4–8 bp DNA sequences. Novel REases are being located by both sequencing of entire genomes followed by bioinformatics analyses and candidate coding genes’ cloning, and a more traditional approach—biochemical screening of bacterial cell lysates.

Type II RM have also been used to identify pathogenic bacteria, such as *Helicobacter pylori*, since they are highly diversified between strains. It has been shown that a total of 22 RM systems with 18 specificities are found in six *Helicobacter pylori* strains and it has been concluded that RM-based methylation patterns of chromosomal DNA may serve as a new typing system to discriminate *Helicobacter pylori* isolates for clinical purposes (Xu et al. 2000; Vale and Vitor 2007). Thus, this paper has two aims: to serve as an aid in the identification of insect-infecting *M. morganii* and to extend the availability of REases for DNA manipulation purposes.

## Results and discussion

### Identification of the restriction endonuclease MmoSTI

A *M. morganii* strain has been isolated from a tropical *A. selene* moth’s larva, exhibiting 100 % deadly infection symptoms. In a recent work we have shown that the disease is caused by a mixed baculovirus-*M. morganii* infection (Skowron et al. 2015). As part of the bacterial strain’s characterization, a lysate was prepared and tested for DNA restriction activity.

Crude lysate was subjected to phosphocellulose chromatography and the restriction activity of effluents was determined. The highest REase activity was observed in fractions with a NaCl concentration of 400–500 mM and just one chromatographic step was sufficient to obtain high quality DNA digests. Therefore, clear, relatively unobscured by non-specific nucleases restriction activity and accompanying REases displaying different specificities was detected in the crude *M. morganii* extract. The detected site-specific REase has been named MmoSTI, according to current nomenclature (Roberts et al. 2004). The overall production of MmoSTI was estimated by serial dilutions as exceeding app. 2000 units/g cells (not shown). Prolonged incubations resulted in clear digestion patterns, indicating low non-specific nucleases content (not shown).

A series of DNA cleavage reactions by the MmoSTI revealed an optimum reaction temperature of 30 °C, activity within a wide range of salt concentration from low through medium to high buffer, with optimum at medium salt buffer (not shown). Determination of the recognition sequence and cleavage site was carried out by three different methods: (*i*) cleavage pattern analysis of short PCR substrates with a few or isolated putative site(s), (*ii*) run-off sequencing of digestion products and (*iii*) shotgun cloning. An additional, general methodological advantage of this combined approach is that it enables the determination of REases’ recognition/cleavage sites even when analyzing partial digests. This is beneficial when analyzing low-concentration enzyme preparations, fast screening for REases or those REases which inherently yield partial digests, regardless of whether an excess of the enzyme is used, e.g., some sub-type IIG REases ( Zylicz-Stachula et al. 2002).

### Recognition sequence estimation by restriction pattern analysis

**Fig. 1:**
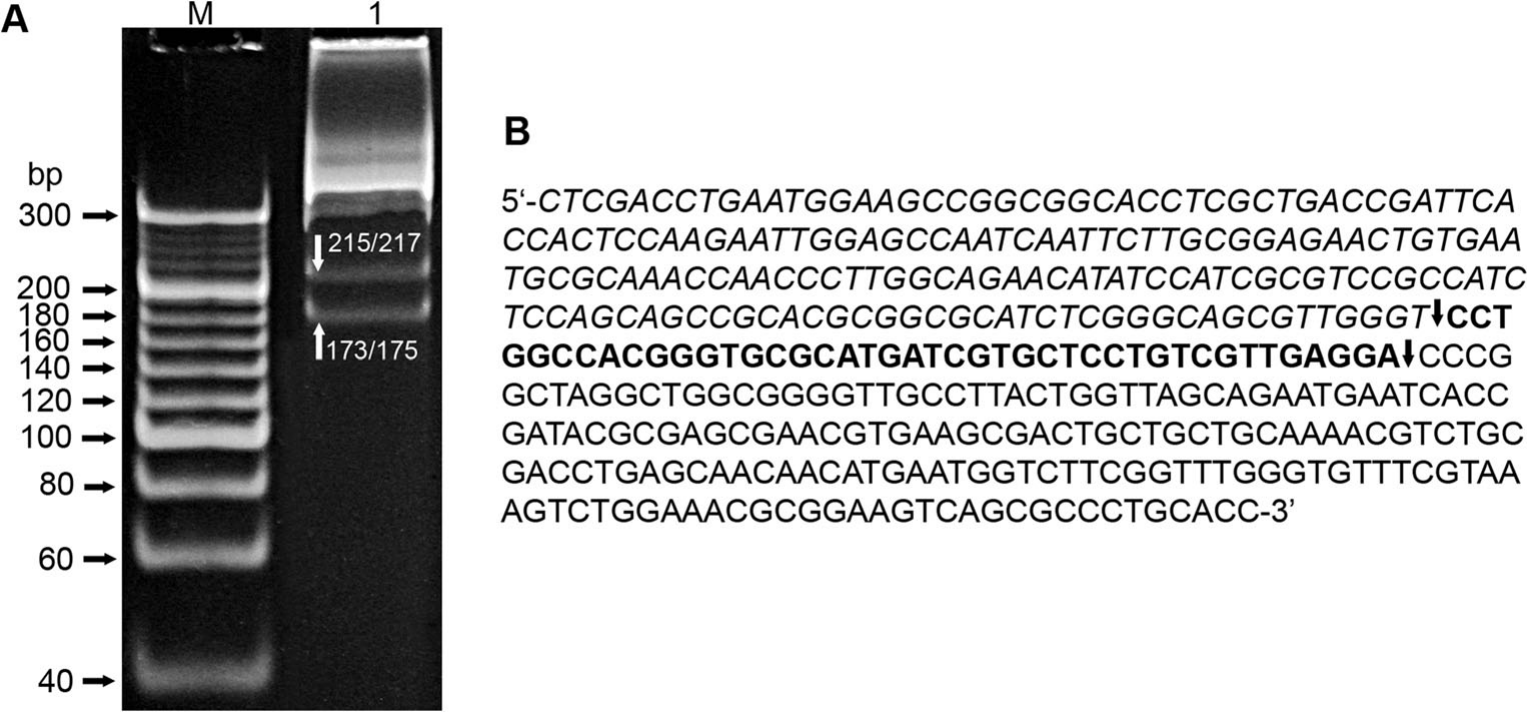
Digestion pattern of a 390 bp PCR substrate cleaved by MmoSTI. **a** 12 % polyacrylamide/TBE PAGE. Lane M, molecular weight marker—O’RangeRuler 20 bp DNA Ladder, Ready-to-Use, Thermo Scientific; lane 1, MmoSTI-digested 390 bp PCR substrate. *White arrows* indicate the generated DNA bands and their corresponding length in bp. **b** DNA sequence of the 390 bp PCR substrate. Three restriction fragments generated by MmoSTI are marked: *italics*—175 bp, *bold*—42 bp, and *regular*—173 bp. *Arrows* indicate cleavage sites of MmoSTI

**Fig. 2:**
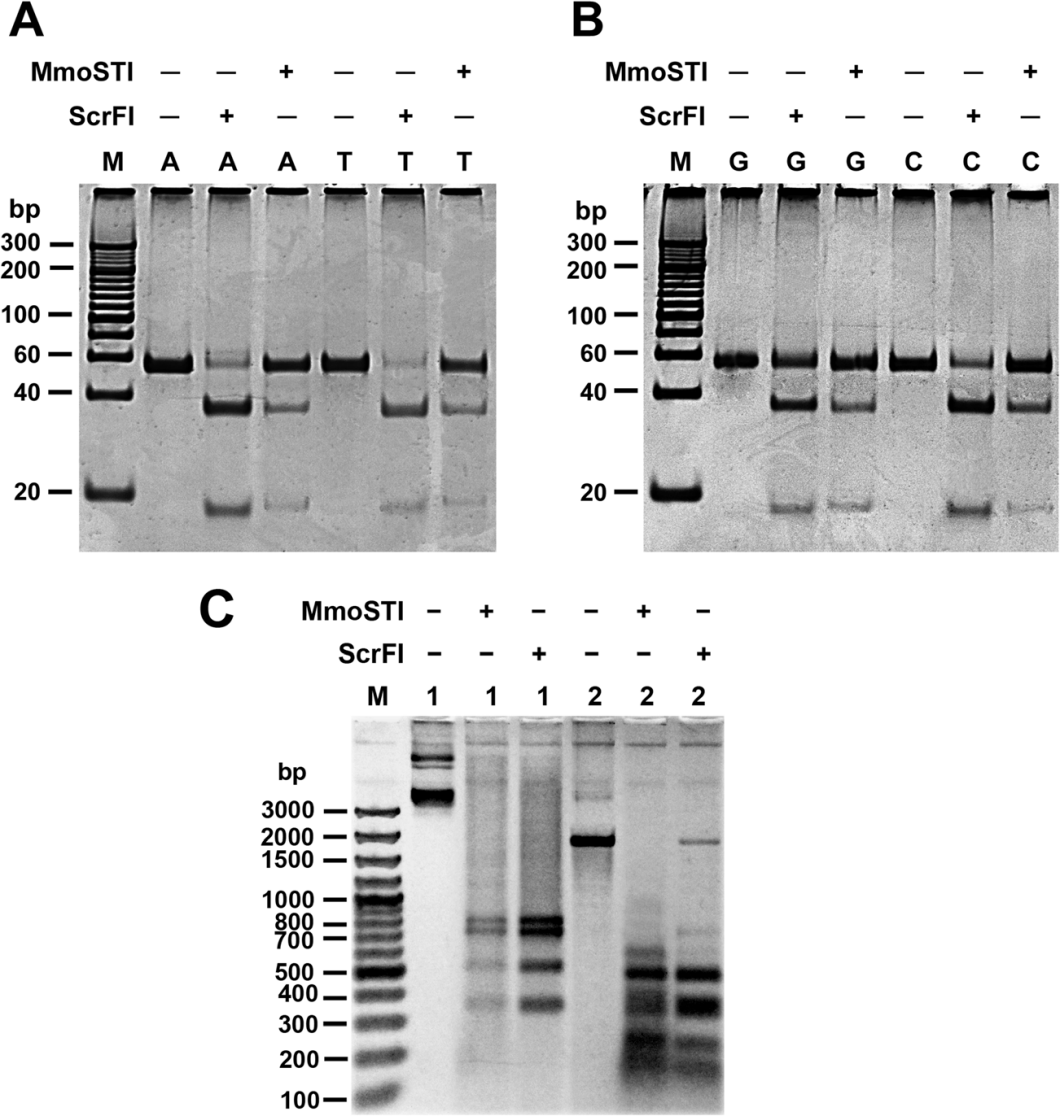
Comparative digestions by MmoSTI and ScrFI. **a** 15 % polyacrylamide/TBE PAGE: Digestions of 54 bp PCR substrate variants containing the sites 5′-CCAGG-3′ or 5′-CCTGG-3′. Lane M, O’RangeRuler 20 bp DNA Ladder, Ready-to-Use, Thermo Scientific; lanes A, uncut 5′-CCAGG-3′ substrate, ScrFI cut 5′-CCAGG-3′ substrate, MmoSTI cut 5′-CCAGG-3′ substrate; lanes T, uncut 5′-CCTGG-3′ substrate, ScrFI cut 5′-CCTGG-3′ substrate, MmoSTI cut 5′-CCTGG-3′ substrate. **b** 15 % polyacrylamide/TBE PAGE: digestions of 54 bp PCR substrate variants containing the sites 5′-CCGGG-3′ or 5′-CCCGG-3′. Lane M, O’RangeRuler 20 bp DNA Ladder, Ready-to-Use, Thermo Scientific; lanes G, uncut 5′-CCGGG-3′ substrate, ScrFI cut 5′-CCGGG-3′ substrate, MmoSTI cut 5′-CCGGG-3′ substrate; lanes C, uncut 5′-CCCGG-3′ substrate, ScrFI cut 5′-CCCGG-3′ substrate, MmoSTI cut 5′-CCCGG-3′ substrate. **c** Agarose gel electrophoresis (1.5 %/TBE) of digested pUC19 and pACYC184-derived 1789 bp PCR substrate. Lane M, O’RangeRuler 20 bp DNA Ladder, Ready-to-Use, Thermo Scientific. Lanes 1, uncut pUC19 DNA, MmoSTI cut pUC19, ScrFI-cut pUC19; lanes 2, uncut 1789 bp PCR substrate, MmoSTI cut 1789 bp PCR, ScrFI-cut 1789 bp PCR

Since MmoSTI is a frequent cutter, short PCR substrates were used for restriction pattern analysis instead of standard longer DNAs, such as plasmids or bacteriophage genomes. Digestion of a 390 bp PCR substrate ( Zylicz-Stachula et al. 2011) by MmoSTI gave two distinct DNA bands in PAGE analysis (Fig. 1a). Basing on the length of the fragments (between 170 and 220 bp), over ten candidate palindromes and interrupted palindromes were located around MmoSTI cleavage points within the 390 bp sequence. Comparing the run-off sequencing results of pUC19 digestion products, the sites 5′-CCTGG-3′ and 5′-CCCGG-3′ were selected for further analysis. Cleavage of the 390 bp PCR substrate cleavage should generate three fragments: 42, 173, and 175 bp (Fig. 1b). Fragments 173 and 175 bp would be visible as one DNA band, as the length difference is below PAGE resolution. The presence of only two restriction fragments after PAGE was due to partial cleavage, as the enzyme obtained was not concentrated. In this case, 173 and 217 as well as 175 and 215 bp fragments were generated, with 173 and 175 bp fragments visible as one and 215 and 217 bp as another band, which collaborates with the pattern obtained (Fig. 1). To evaluate all potential variants of the MmoSTI cognate site with respect to the 3rd variable base, four 54 bp PCR substrates, differing only in a single bp within asymmetrically located 5′-CCNGG-3′ sequences, were generated. The digestion products would be 33 and 16 bp in each case, if all variants are cleaved. As shown in Fig. 2a, b, all 5′-CCNGG-3′ are cleaved both by MmoSTI and the prototype ScrFI. To finally validate this analysis, a comparative digestion of the pUC19 plasmid, as well as a long 1789 bp PCR substrate (Krefft et al. 2015), with MmoSTI and ScrFI was conducted and resulted in the same digestion patterns. As both enzymes cleave DNA frequently, the obtained multiple bands are grouped within a small fragment size range (Fig. 2c).

### Determining the MmoSTI recognition sequence and cleavage site by run-off sequencing of pUC19/MmoSTI digestion products

**Fig. 3:**
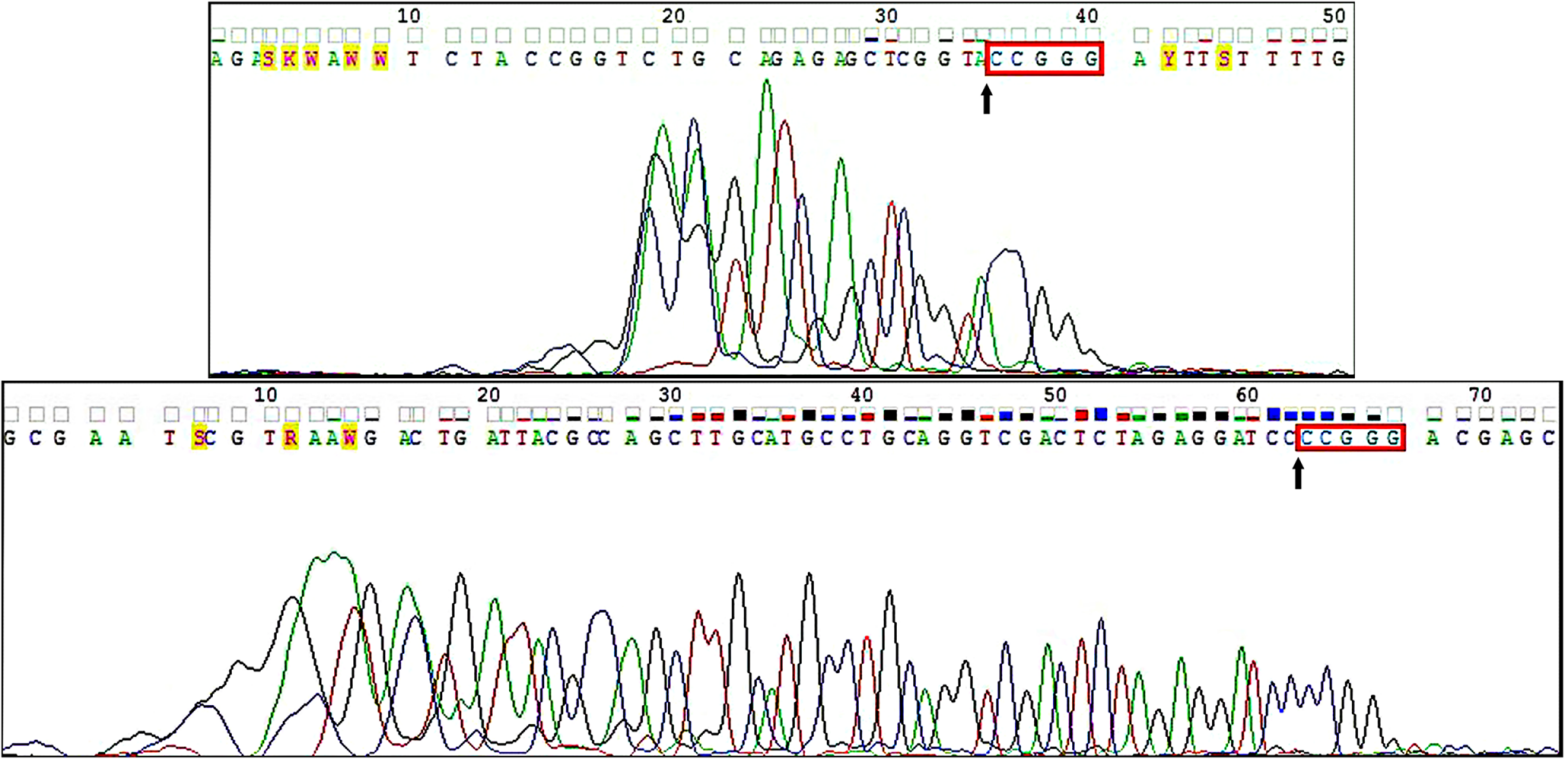
Run-off sequencing read showing MmoSTI cleavage site in pUC19. *Top*: forward primer, *bottom*: reverse primer; *box*: recognition site of MmoSTI; *arrows* indicate cleavage site

In run-off sequencing, DNA polymerase extends DNA strands to the point where it falls off the end of the template. The sudden stop in sequencing peaks obtained from the forward primer shows the recognition site of MmoSTI: 5′-CCGGG-3′ and the cleavage point being before the first C (Fig. 3). In the case of the sequencing read generated by the reverse primer, the last peak representing G is smaller than previous peaks, but clearly stands out from the background. Such artefacts at the ends of DNA sequencing are frequently observed, as polymerases have difficulties in the last base incorporation efficiency, whereas capillary electrophoresis is also of lower resolution with a terminal stretch of identical bases. The sequencing results indicate that the last five nt of the cut fragment are 5′-CCGGG-3′ or 5′-CCCGG-3′, thus the MmoSTI recognition site is an interrupted palindrome, with a variable internal 3rd base.

### MmoSTI recognition and cleavage sequence determination by shotgun cloning and sequencing

Fragments of λ and *E. coli* genomes digested with MmoSTI were blunted with T4 DNA polymerase/dNTPs, ligated into the SmaI site of pUC19 and insert junctions were sequenced. The clones’ sequencing results provided independent confirmation of the MmoSTI putative recognition site. Further analysis considered both the potential MmoSTI recognition sites present in the inserts and DNA forming the clipped off flanking regions. Table 1 shows λ or *E. coli* genome fragments with MmoSTI recognition sequences marked.

**Table 1:**
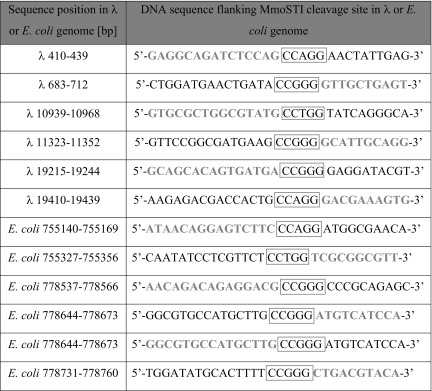
Determination of the MmoSTI recognition sequence and cleavage site by shotgun cloning and sequencing of MmoSTI restriction fragments

Base numbering refers to the 5′ → 3′ orientation of λ or *E. coli* genome. Grey and emboldened, terminal part of restriction fragment generated by MmoSTI, blunted with T4 DNA polymerase/dNTPs and cloned into pUC19. Black, λ or *E. coli* genome sequence adjacent to the cloned restriction fragment. Grey box, MmoSTI recognition sequence

These results show that the recognition site of MmoSTI is 5′-CCNGG-3′. The fact that the entire recognition sequence is within the filled-in inserts indicates that MmoSTI cleaves DNA leaving 5-nt long, ss, 5′- cohesive ends. Thus, MmoSTI, recognizing an interrupted palindrome, is a subtype IIP REase (Loenen et al. 2014) exhibiting high cleavage frequency.

Current REBASE data (04/06/2015) indicates that the ScrFI prototype enzyme from *Streptococcus cremoris* F recognizes a 5′-CC|NGG-3′ sequence and cleaves after the second C of the recognition site, leaving 1-nt protruding 5′ ss DNA termini. The REase has 129 isoschizomers and neoschizomers, found either through biochemical analyses of bacterial cellular extracts or as putative genes, detected using genomic bioinformatics. Most originate from bacteria with a relatively narrow range of optimal temperature—mezophiles growing between 26 and 37 °C, however, there are some present in psychrophiles (0 °C) and thermophiles (55 °C). MmoSTI was isolated from *M. morganii* ST with an optimal growth temperature of 30–37 °C. Out of all known ScrFI isoschizomers, 97 comprise putative ORFs, predicted to code for REases, recognizing 5′-CCNGG-3′ sites, without experimental validations and with no estimates of cleavage positions. Four isoschizomers cleave recognition sites like the prototype, ScrFI, whereas 12 neoschizomers cleave before the first C, leaving long 5-nt protruding 5′ ss DNA termini and no isoschizomer cleaving before the second C was found (REBASE 04/06/2015). Thus far, no REases have been found and characterized biochemically in *M. morganii*. However, several putative MTase-coding genes, with predicted 5′-GATC-3′ and two with predicted 5′-CCNGG-3′ specificities of coded MTases, (M.MmoKTORF102P and M.MmoSC01ORF3004P, REBASE 04/06/2015) were found in genomes of *M. morganii*.

To conclude, the discovered REase can serve for two main purposes: *i)* aid in pathogenic bacterial strain identification and *ii)* provide a high frequency cutting REase for DNA manipulations, which can be extracted from an easily cultured bacterium, *M. morganii*, using a simple purification protocol.

## Materials and methods

### Bacterial strains, DNAs, reagents

*Escherichia coli* (*E. coli*) DH11S [*mcr*A ∆(*mrr*-hsd RMS-*mcr* BC) ∆(*lac*-*pro*AB) ∆(*rec*A 1398) *deo*R, *rps*L *srl-thi*, *sup*E/F’, *pro*AB-*lac*I^q^ Z ∆M15] was from Novagen / Merck KGaA (Darmstadt, Germany). *M. morganii* was isolated from *A. selene* larva cultivated in Stratford Butterfly Farm (Stratford-Upon-Avon, UK). Plasmid pUC19, *E. coli* DNA and λ cI857ts were isolated according to Sambrook et al. 1989. Marathon Taq DNA Polymerase was from A&A Biotechnologies (Gdansk, Poland), Oligonucleotide synthesis was performed at Genomed (Warsaw, Poland). DNA, protein markers and the Miniprep DNA purification kit were from Thermo Scientific (Fermentas, Vilnus, Lithuania). All other reagents were purchased from Sigma-Aldrich (St Louis, MO, USA).

### Screening for DNA restriction activity

Cuticle surfaces of infected *A. selene* larvae were imprinted and streaked on agar plates with LB medium (Sambrook et al. 1989) and incubated for 48 h. The dominant strain, as a potential etiological agent, was further studied, which included colony morphology description, Gram staining, antibiotic resistance determination, growth temperature optimization and identification through mass spectrometry (Skowron et al. 2015). Bacterial lysate was prepared using a single bacterial colony grown at 30 °C on LB plates, which was introduced into 20 ml of LB and incubated overnight at 30 °C in a shaker. Subsequently, 1000 ml of LB was inoculated, grown with vigorous aeration until the culture’s entrance into the stationary phase, chilled down and centrifuged. The bacterial pellet was suspended in phosphocellulose chromatography buffer (50 mM K/PO4 pH 7.2, 0.5 mM EDTA, 50 mM NaCl, 0.05 % Triton X-100, 5 mM β-mercaptoethanol, 0.5 mM PMSF, 1/4 Sigma tablet protease inhibitors, containing AEBSF, aprotinin, bestatin, EDTA, E-64, leupeptin). Lysozyme was added to 0.5 mg/ml and the suspension was incubated in ice for 1 h, sonicated, and centrifuged. The obtained crude lysate was subjected to phosphocellulose chromatography. Crude lysate and chromatography fractions were assayed for restriction activity by incubation with 500 ng of substrate DNA from 1 h to overnight at 30 °C in 25 μl reactions in medium salt buffer (50 mM NaCl, pH 7.5, 10 mM MgCl_2_; crude lysate additionally supplemented with 50 μg/ml of RNase A). Reactions determining the recognition/cleavage specificity of MmoSTI were carried out in the same conditions. The non-specific nuclease content was estimated through long incubations (4 h to overnight) of serial dilutions of the purified enzyme with pUC19 DNA in medium salt buffer and assessment of the digestion patterns’ clarity.

### Determination of MmoSTI recognition and cleavage sites

#### Run-off sequencing of digestion products

Plasmid pUC19 was digested overnight at 30 °C with MmoSTI. Proteins were removed by proteinase K digestion, phenol/chloroform extracted and ethanol precipitated. Purified cleaved products were subjected to Sanger automated sequencing at Genomed (Warsaw, Poland) using standard pUC19 primers.

#### Cleavage pattern analysis of PCR substrates

A set of custom PCR substrates was prepared (Figs. 1 and 2). The 390 bp substrate was amplified with sequence-modifying primers from a pBR322 template ( Zylicz-Stachula et al. 2011) and the 1789 bp substrate was amplified from the pACYC184 plasmid (Krefft et al. 2015). Four variants of the 54 bp substrate, differing in a single bp within isolated 5′-CCNGG-3′ sites, were prepared as follows: four 54 nt ssDNAs were synthesized and used as a PCR template (MmoSTI putative recognition sites in bold, underlined): 5′-GGGCGCCATCTCGACCGAAGAGAGGCCAAGCAT**CCAGG**GCCGCGCTGCGGACCC-3′, 5′-GGGCGCCATCTCGACCGAAGAGAGGCCAAGCAT**CCTGG**GCCGCGCTGCGGACCC-3′, 5′-GGGCGCCATCTCGACCGAAGAGAGGCCAAGCAT**CCCGG**GCCGCGCTGCGGACCC-3′, 5′-GGGCGCCATCTCGACCGAAGAGAGGCCAAGCAT**CCGGG**GCCGCGCTGCGGACCC-3′. The amplification was conducted with primers: 5′-GGGCGCCATCTCGACC-3′ and 5′-GGGTCCGCAGCGCGGCC-3′ in 100 μl reactions, containing 1x Marathon PCR buffer, 0.4 mM of each dNTP, 0.5 μM of each primer, 100 ng template, and 1 unit of Marathon DNA polymerase. The hot-start PCR profile included 32 cycles of 95, 50, and 68 °C. Substrates were purified on 1.8 % agarose/TBE gels, electroeluted, phenol/chloroform extracted and ethanol precipitated. MmoSTI-digested PCR substrates were treated with proteinase K, phenol/chloroform extracted, ethanol precipitated and subjected to PAGE analysis.

#### Shotgun cloning

1.5 μg of λ and *E. coli* DNA were digested by MmoSTI at 30 °C for 3 h. Cohesive DNA ends were filled by T4 DNA polymerase/dNTPs. DNA was purified through phenol/chloroform extraction and ethanol precipitated. Resulting DNA fragments were cloned into the SmaI site of pUC19 vector and electrotransformed into *E. coli*. Bacteria were plated onto X-Gal/IPTG plates (α-complementation screening). Nineteen selected clones were used directly as a template in PCR with the standard primers: 5′-CGCCAGGGTTTTCCCAGTCACGAC-3′ and 5′- AGCGGATAACAATTTCACACAGG-3′. Recombinant bacteria were cultured in LB and plasmids were isolated through alkaline lysis, phenol/chloroform extracted, ethanol precipitated, and subjected to Sanger sequencing.
